# How does former Salter innominate osteotomy in patients with Legg–Calvé–Perthes disease influence acetabular orientation? An MRI-based study

**DOI:** 10.1093/jhps/hnab063

**Published:** 2021-08-21

**Authors:** Petri Bellova, Jens Goronzy, Sophia Blum, Simon Bürger, Albrecht Hartmann, Klaus-Peter Günther, Falk Thielemann

**Affiliations:** Department of Orthopedics, Trauma and Plastic Surgery, University Hospital Carl Gustav Carus, TU Dresden, Fetscherstr. 74, Dresden 01307, Germany; Department of Orthopedics, Trauma and Plastic Surgery, University Hospital Carl Gustav Carus, TU Dresden, Fetscherstr. 74, Dresden 01307, Germany; Department of Radiology, University Hospital Carl Gustav Carus, TU Dresden, Fetscherstr. 74, Dresden 01307, Germany; Faculty of Medicine, TU Dresden, Helmholtzstr.10, Dresden 01069, Germany; Department of Orthopedics, Trauma and Plastic Surgery, University Hospital Carl Gustav Carus, TU Dresden, Fetscherstr. 74, Dresden 01307, Germany; Department of Orthopedics, Trauma and Plastic Surgery, University Hospital Carl Gustav Carus, TU Dresden, Fetscherstr. 74, Dresden 01307, Germany; Department of Orthopedics, Trauma and Plastic Surgery, University Hospital Carl Gustav Carus, TU Dresden, Fetscherstr. 74, Dresden 01307, Germany

## Abstract

Salter innominate osteotomy (SIO) has been successfully used in the treatment of Legg–Calvé–Perthes disease (LCPD). Recent studies that have raised concerns about acetabular retroversion after SIO have been based on plain radiographs. In order to assess the true acetabular orientation, the present study uses a specific magnetic resonance imaging (MRI) technique. In addition, the association between acetabular morphology and clinical function as well as health-related quality of life was assessed. Twenty-three patients with 24 operated hips who underwent SIO for LCPD between January 2004 and November 2014 were included. Mean age was 8.5 ± 2.2 years at surgery and 18.5 ± 2.9 years at follow-up. MRIs were conducted at 1.5 T using radial sequences. The analysis included the acetabular version, acetabular sector angles (ASAs) and alpha angles. Plain radiographs were used in order to obtain the Stulberg classification. Patient-related outcome measures included the international Hip Outcome Tool and Euroqol-5 dimensions scores. In comparison to the non-operated side, the MRI of previously operated hips showed no difference of version at the center of the femoral head but significantly decreased version just below the roof level. As a marker for posterior acetabular coverage, the ASAs between 9 and 11 o’clock were significantly decreased when compared with non-operated hips. In hips with a mild acetabular retroversion (<15°), the function was significantly decreased when compared with non-retroverted hips. The SIO is an effective tool in order to restore acetabular containment in LCPD. When compared with the non-operated hips, our collective displays only moderate changes of acetabular orientation and coverage.

## INTRODUCTION

Legg–Calvé–Perthes disease (LCPD) is a common hip pathology during early childhood that affects mainly boys and leads to hip pain and limitation of the range of motion. The impaired circulation of the femoral head is followed by a collapse of the bone structure of the proximal femoral epiphysis with potential secondary deformation and degenerative changes [1].

The primary treatment goal in LCPD patients is to restore containment of the hip joint [2]. This can be achieved by conservative as well as surgical measures. Among other osteotomies, the Salter innominate osteotomy (SIO) [3, 4] is an established procedure in the treatment of LCPD patients [4–8]. If adequate containment of the hip is achieved, a remodeling to a congruent shape is possible even in the face of collapse and deformation [5, 9].

Several studies have reported on the results of former SIO [8–13]. While these studies have mostly displayed good long-term results, concerns have been raised about potential acetabular retroversion due to overcorrection. Acetabular retroversion might lead to femoroacetabular impingement [14], which is a recognized risk factor for secondary hip osteoarthritis (OA) [15].

Studies on this topic have mainly used conventional radiographs in order to determine the acetabular version. The findings on radiographs, however, may be highly biased by pelvic tilt and other inherent limitations of conventional radiographs [16–19].

Magnetic resonance imaging (MRI) has the capacity of providing accurate measurements without radiation exposure [20]. In our literature review, we found two studies that assessed the sequelae of LCPD by using three-dimensional imaging [21, 22], while the respective patients underwent different types of treatment. However, we found no study that investigated the effects of the SIO by using MRI during the follow-up (FU).

Therefore, our aim was to determine the three-dimensional acetabular morphology by MRI in skeletally mature LCPD patients who had undergone SIO during childhood. Furthermore, MRI-based coverage and version were compared with standard radiographic signs, while these parameters were also related to functional scores.

## MATERIALS AND METHODS

### Study population

**Fig. 1.**
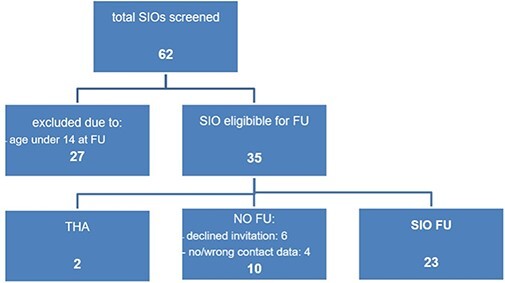
Strengthening the Reporting of Observational Studies in Epidemiology (STROBE) diagram of operated hips (flowchart of patient inclusion) FU, follow-up; SIO, Salter Innominate Osteotomy; THA, total hip arthroplasty.

Sixty-two consecutive patients had undergone SIO with and without intertrochanteric varisation osteotomy due to LCPD in our University Center between January 2004 and November 2014. Exclusion criteria for this retrospective cohort study (Level 3) were any neuromuscular disorder, an open triradiate cartilage, an age below 14 years at FU and an inability to undergo MRI or to follow the physician’s instructions due to cognitive impairment. As 27 patients were too young to have a closed triradiate cartilage and the others did not fulfill any exclusion criteria, 35 patients were eligible for FU. Two of them had already undergone total hip replacement and 10 patients could not be investigated due to missing contact data (*n* = 4) or refusal to participate (*n* = 6). Finally, 23 patients were available for the final FU and MRI investigation (Fig. 1). Approval was obtained by the local Ethics Committee.

All surgeries were performed by F.T. and K-P.G. Twenty-two patients received surgery on one hip while one patient received bilateral surgery. As each hip was treated as an independent data point, data from 24 operated hips and 22 non-operated hips were pooled together, and the respective mean values were compared against each other. All non-operated hips were once again reviewed at FU and were found to be free of LCPD and of pain.

### Follow-up (clinical)

FU clinical examination included the assessment of the anterior and posterior impingement sign.

Regarding patient-related outcome measures (PROMs), we chose the international Hip Outcome Tool (iHOT) [23] as a score especially developed for joint preserving surgery. Furthermore, the Euroqol-5 dimensions (EQ-5D) [24] score was obtained as a measure for health-related quality of life.

### Follow-up (conventional radiographs)

Conventional radiographic evaluation consisted of a standard antero-posterior (AP) pelvic radiograph in the supine position as well as a frog-leg lateral hip radiograph on the operated side. On AP radiographs, lateral center-edge (LCE) angles [25] and acetabular indices (AIs) [26] were measured for both the operated and the non-operated hips. Furthermore, the crossover sign as well as the ischial spine sign were determined [16]. On frog-leg lateral radiographs, alpha angles were determined [27].

All operated hips were classified according to the Stulberg (SB) classification [28] using conventional AP radiographs.

SB classes I and II were pooled into one group (spherical head type = SHT), as were SB classes III, IV and V (deformed head type = DHT).

Furthermore, hips were graded according to the Kellgren and Lawrence (KL) classification for OA [29].

### Follow-up (MRI)

Non-contrast MRI was performed and measured solely for study purposes using a specified technique with a high inter- and intraobserver reliability by one observer (J.G.) [20]. In order to compensate for pelvic obliquity and rotation, the centers of the reformation axis were aligned with the centers of the femoral heads in both the transverse and coronal planes. MRI-based comparison of operated and non-operated hips could be performed in 20 out of 24 hips. In four hips, an analysis was not possible due to metal artifacts of remaining osteosynthesis devices (*n* = 2) and bilateral previous surgery (*n* = 2).

**Fig. 2.**
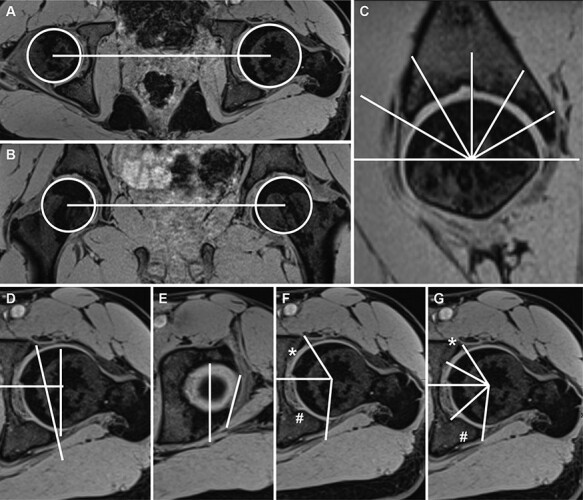
Magnetic resonance imaging (MRI) pelvic alignment and performed measurements: MRI alignment in the (A) axial, (B) coronal and (C) sagittal planes presenting the measured clockwise plains; (D) measurement of the anteversion at the femoral head center, (E) the anteversion at the acetabulum roof, (F) the anterior (*; 3 o’clock) and posterior (#; 9 o’clock) Acetabular Sector Angles (ASA) and (G) the Cartilage Covered Area Angles (CCAA) anteriorly (*; 3 o’clock) and posteriorly (#; 9 o’clock).

Modified acetabular sector angles (ASAs) were measured in a clockwise manner from 9 to 3 o’clock (9/10/11/12/1/2/3 o’clock) (Fig. 2). Furthermore, the cartilage covered area angle (CCAA) between the acetabular edge and the acetabular fossa as well as the alpha angles were measured accordingly. The latter was measured using the most approximated circle based on the femoral head morphology. The acetabular version was measured in the transverse plane passing through the center of the femoral head [30] as well as just below the roof level when the most cranial contour of the femoral head became visible for the first time.

### Statistical analysis

Statistical analysis was performed using SPSS (IBM, V. 25, Chicago, IL, USA).

The first comparisons were drawn between the overall collective of operated and non-operated hips. Furthermore, the subgroup analyses included a comparison between (i) the SHT and DHT hips on the operated side, (ii) the SHT hips and non-operated hips and (iii) the DHT hips and non-operated hips. In order to preclude anatomical differences within the control group, non-operated hips in the SHT and DHT groups were compared, and no significant differences were found. Continuous variables were presented as the mean with standard deviation (SD) and the range, and categorical variables were presented as counts and percentages. Between-group comparisons were performed using paired t-tests, Mann–Whitney U tests or Wilcoxon signed rank tests (when applicable). The significance level was set at *P* < 0.05.

## RESULTS

Eight patients had surgery on their right hip and 14 on their left hip. Twenty patients were male (87.0%) and three were female. Further baseline data are displayed in Table I.

**Table I.** Baseline characteristics of patients in the different groups^a^

		*Total*	*SHT*	*DHT*
Age at surgery		8.5 ± 2.2 (4.6–13.7)	7.7 ± 1.3 (5.5–9.7)	9.0 ± 2.5 (4.6–13.7)
Femoral osteotomy		20/24 (83.3%)	9/9 (100%)	11/15 (73.3%)
Age at follow-up		18.5 ± 2.9 (14.3–24.1)	17.3 ± 3.3 (14.3–24.0)	19.2 ± 2.6 (15.2–24.2)
SB classification	I	6 (25.0%)	6 (66.7%)	
	II	3 (12.5%)	3 (33.3%)	
	III	11 (45.8%)		11 (73.3%)
	IV	3 (12.5%)		3 (20.0%)
	V	1 (4.2%)		1 (6.7%)

a SHT (SB I–II); DHT (SB III–V).

DHT, Deformed Head Types; SB, Stulberg; SHT, Spherical Head Types.

There were no differences between the SHT and DHT subgroups both concerning age at surgery and at FU (*P* = 0.558 and *P* = 0.064, respectively).

A positive anterior as well as posterior impingement sign occurred more often on the operated side than on the non-operated side (*P* = 0.003 and *P* = 0.02, respectively). Differences between the subgroups are shown in Table II. iHOT and EQ-5D score values were similar among the subgroups (*P* = 0.123 and *P* = 0.73, respectively).

**Table II.** Outcome scores and clinical examination at follow-up in all operated hips and contralateral non-operated hips^a^

	*Total*	*SHT*	*DHT*	*Non-operated*
iHOT	80.7 ± 16.3 (39.2–95)	85.2 ± 13 (62.9–95)	77.8 ± 17.9 (39.2–94.2)	–
EQ-5D	85 ± 14.5 (60–100)	88.4 ± 15 (60–100)	82.8 ± 14.2 (50–100)	–
Anterior impingement sign (%)	12/22 (54.5) ‡	4/9 (44.4)	8/13 (61.5) ‡	3/22 (13.6)
Posterior impingement sign (%)	8/22 (36.4) ‡	6/9 (66.7)	5/13 (38.5)	1/22 (4.5)

a Values of continuous parameters are given as mean ± SD with range in parentheses. Categorical variables are presented as counts with percentages;

† significant difference when compared with DHT (SB III–V) subgroup;

‡ significant difference when compared with non-operated side;

* significant difference between SHT (SB I–II) and DHT (SB III–V) in non-operated group.

DHT, Deformed Head Types; EQ-5D, Euroqol-5-dimensions; iHOT, international Hip Outcome Tool; SHT, spherical head types.

Pelvic radiographs at FU showed a decreased lateral coverage, as the LCE angle of all operated hips was lower than that of non-operated hips (*P* = 0.009). The AI was similar between the two (*P* = 0.545). Subgroup differences are presented in Table III.

**Table III.** Radiographic measurements at follow-up in all operated hips and contralateral non-operated hips^a^

		*Total*	*SHT*	*DHT*	*Non-operated*
LCE angle		25.8 ± 8.1 (10.0–43.9)‡	30.0 ± 4.9 (19.7–36.2)†	23.2 ± 8.7 (10.0–43.9)	31.1 ± 6.9 (22.1–46.4)
AI		9.6 ± 7.4 (0.3–29.8)	5.4 ± 3.0 (0.3–8.8)†	12.3 ± 8.2 (1.4–29.8)	7.7 ± 6.4 (−3.8–18.0)
Alpha angle		62.6 ± 28.9 (33.0–134.0)	50.4 ± 8.7 (40.6–61.0)	72.0 ± 35.7 (33.0–134.0)	–
Hips with crossover sign (%)		18/24 (75.0)	6/9 (66.7)	12/15 (80.0)	11/21 (52.4)
Hips with ischial spine sign ()		19/24 (79.2)‡	8/9 (88.9)‡	11/15 (73.3)	12/24 (50.0)
Osteoarthritis KL (%)	0	4 (16.7)	3 (33.3)	1 (6.7)	15 (62.5)
	1	19 (79.2)	6 (66.7)	13 (86.7)	9 (37.5)
	2	1 (4.2)	–	1 (6.7)	–

a Values of continuous parameters are given as mean ± standard deviation (SD) with range in parentheses;

† significant difference when compared with DHT subgroup;

‡ significant difference when compared with non-operated side;

* significant difference between SHT and DHT in non-operated group. SHT (SB I–II); DHT (SB III–V).

AI, Acetabular Index; DHT, Deformed Head Types; LCE, Lateral Center Edge; KL, Kellgren&Lawrence; SHT, Spherical Head Types.

Nearly all hips showed no or doubtful signs of OA (KL grades 0 and 1), and only one hip was classified as KL grade 2 (Table I).

The version just below the acetabular roof was negative on average (<0°) for both the operated and non-operated hips and was significantly lower for the operated hips (*P* = 0.006). Especially, SHT hips were shown to have a decreased version both in comparison with DHT hips and with the control group.

Hips with SIO had significantly lower ASAs in the 9, 10, 11 as well as 3 o’clock positions in comparison with the non-operated hips (*P* = 0.001, *P* < 0.001, *P* = 0.011 and *P* = 0.004, respectively). The subgroup differences as well as the assessment of CCAA angles are displayed in Table IV.

**Table IV.** MRI measurements at follow-up in all operated hips and contralateral non-operated hips^a^

		*Total*	*SHT*	*DHT*	*Non-operated*
Anteversion acetabulum roof		−6.8 ± 6.7 (−22.5–7.9) ‡	−11.5 ± 5.7 (−22.5-(−4.5) †‡	−4.2 ± 5.9 (−10.9–7.9)	−1.8 ± 7.9 (−17.8–9.2)*
>0° (%)		4/22 (18.2)	0/8	4/14 (28.6)	9/20 (45.0)
Anteversion acetabulum femoral head center		14.5 ± 5.5 (5.7–33.1)	12.4 ± 3.0 (5.7–15.0)	15.7 ± 6.3 (7.0–33.1)	14.8 ± 4.6 (6.6–24.6)
>15° (%)		7/22 (31.8)	1/8 (12.5)	6/14 (42.9)	6/20 (30)
ASA	3	54.1 ± 8.5 (36.6–68.2)‡	59.2 ± 6.4 (51.2–67.6)†	51.2 ± 8.4 (36.6–68.2)‡	61.8 ± 6.8 (50.3–71.4)
	2	87.9 ± 17.5 (51.0–114.0)	97.1 ± 13.3 (82.0–114.0)	82.7 ± 17.8 (51.0–108.0)	91.8 ± 13.4 (69.5–121.0)
	1	117.2 ± 9.3 (99.4–129.0)	122.5 ± 5.6 (109.9–127.0)†	114.1 ± 9.7 (99.4–129.0)	119.4 ± 7.1 (107.6–134.6)
	12	117.7 ± 9.9 (99.7–136.9)	122.9 ± 6.5 (109.0–129.0)	114.7 ± 10.5 (99.7–136.9)‡	123.3 ± 6.6 (109.4–133.2)
	11	109.1 ± 10.5 (88.3–126.7)‡	113.8 ± 6.6 (102.0–122.4)	106.4 ± 11.5 (88.3–126.7)‡	116.9 ± 7.7 (101.7–132.9)
	10	94.9 ± 9.4 (76.0–112.2)‡	97.5 ± 5.5 (91.0–107.6)	93.4 ± 10.9 (76.0–112.2)‡	103.4 ± 7.5 (90.5–117.5)
	9	82.3 ± 8.8 (66.0–97.4)‡	84.7 ± 7.2 (76.0–94.0)‡	80.9 ± 9.6 (66.0–97.4)‡	90.7 ± 6.0 (81.7–106.3)
CCAA	3	31.2 ± 7.5 (16.8–46)	35.3 ± 6.8 (25.8–46)	28.9 ±7.1 (16.8–39.4)	32.7 ± 5.3 (18.7–42.4)
	2	61.8 ± 14.4 (33–60)	62.5 ± 11.2 (46–75.2)	61.4 ± 16.4 (33–90)	58.4 ± 11.8 (44.4–84.1)
	1	77.7 ± 16.0 (44.8–107.6)	84.5 ± 13.2 (70.0–104.0)	73.9 ± 16.6 (44.8–107.6)	78.4 ± 8.0 (60.0–94.1)
	12	68.1 ± 17.4 (32.5–113.3)	69.8 ± 10.0 (51.7–84.0)	67.1 ± 20.8 (32.5–113.3)	69.3 ± 11.4 (52.1–89.2)
	11	68.0 ± 13.1 (45.0–102.0)	73.6 ± 15.6 (57.6–102.0)	64.8 ± 10.8 (45.0–81.2)‡	73.6 ± 8.3 (59.6–88.2)
	10	56.6 ± 9.1 (41.0–69.5)	54.7 ± 9.6 (42.7–69.0)	57.6 ± 9.0 (41.0–69.5)	59.8 ± 7.1 (39.3–70.0)
	9	47.9 ± 10.6 (29.9–74.4)	47.3 ± 11.1 (29.9–70.0)	48.2 ± 10.6 (37.5–74.4)	53.4 ± 5.5 (38.7–63.0)
Alpha angle	3	46.2 ± 20.9 (28.0–90.0)	40.1 ± 4.5 (32.3–46.0)	50.0 ± 26.1 (28.0–90.0)	42.6 ± 7.0 (32.4–59.3)
	2	64.4 ± 25.2 (34.0–117.9)	52.8 ± 7.7 (41.5–64.6)†	71.6 ± 29.6 (34.0–117.9)	57.4 ± 10.4 (41.8–76.6)
	1	65.9 ± 19.2 (42.3–120.0)	54.3 ± 9.1 (42.3–71.6)†	73.0 ± 20.5 (43.3–120.0)‡	56.0 ± 8.8 (42.0–75.2)
	12	62.6 ± 18.3 (33.5–103.2)‡	47.3 ± 8.0 (33.5–58.0)†	72.1 ± 16.4 (47.4–103.2)‡	48.4 ± 11.9 (34.9–79.6)
	11	52.1 ± 16.0 (34.4–85.1)	42.1 ± 6.2 (34.4–53.4)†	58.2 ± 17.2 (35.5–85.1)‡	42.3 ± 9.7 (33.2–76.3)
	10	42.1 ± 12.8 (20.3–75.0)	41.1 ± 5.8 (32.3–51.1)	42.8 ± 15.8 (20.3–75.0)	39.1 ± 4.8 (30.2–51.3)
	9	32.6 ± 9.4 (18.5–59.7)	29.8 ± 5.3 (23.5–41.5)	34.3 ± 11.1 (18.5–59.7)	34.3 ± 5.8 (23.3–43.1)

a Values of continuous parameters are given in degrees as mean ± standard deviation (SD) with range in parentheses;

† significant difference when compared with Deformed Head Types (DHT) subgroup;

‡ significant difference when compared with non-operated side;

* significant difference between Spherical Head Types (SHT) and DHT in non-operated group. SHT (Stulberg (SB) I–II); DHT (SB III–V).

ASA, Acetabular Sector Angle, CCAA, Cartilage Covered Area Angle; LCE, Lateral Center Edge.

Alpha angles in the SHT subgroup showed no significant differences when compared with the non-operated control group, whereas the alpha angles in the DHT subgroup were increased in multiple positions (Table IV).

**Fig. 3.**
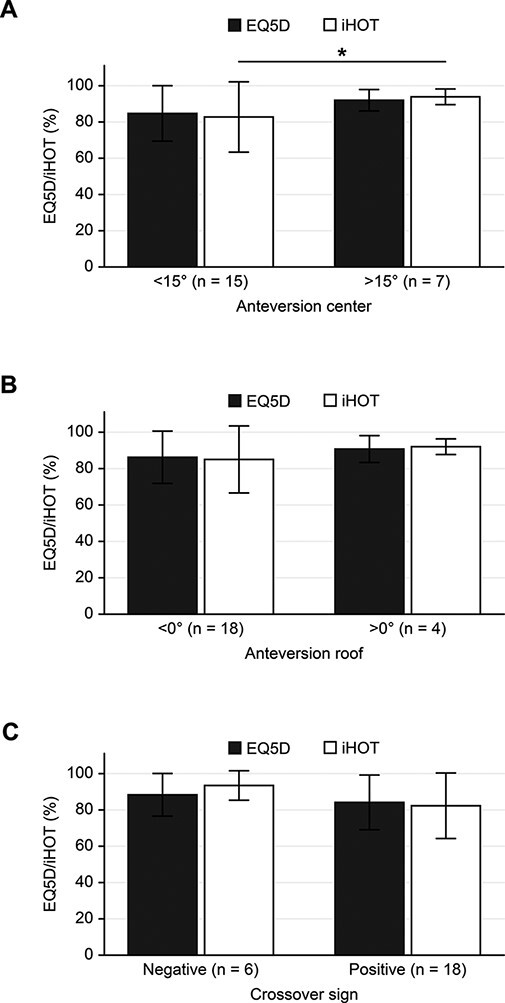
Association between clinical outcome (international Hip Outcome Tool (iHOT) algofunction and health-related quality of life) and magnetic resonance imaging (MRI)-determined acetabular anteversion ((A): at the center level; (B): just below the roof level) as well as (C) radiographically determined crossover sign. Euroqol-5-Dimensions (EQ-5D) (black bars) and iHOT (white bars) are presented as mean with standard deviation (SD) (error bars). Asterisks indicate statistical significance (*P* < 0.05).

Patients with a reduced acetabular version at the femoral head center (<15°) had lower iHOT values than patients with an anteversion >15° (*P* = 0.049), while there was no difference regarding the EQ-5D score. Acetabular roof retroversion (<0°) had no influence on the iHOT and EQ-5D (*P* = 0.967 and *P* = 0.774, respectively). In patients with a positive crossover sign on conventional radiographs, both the iHOT and EQ-5D scores were lower when compared with those with a negative crossover sign, albeit not statistically significant (*P* = 0.05 and *P* = 0.50, respectively) (Fig. 3).

**Fig. 4.**
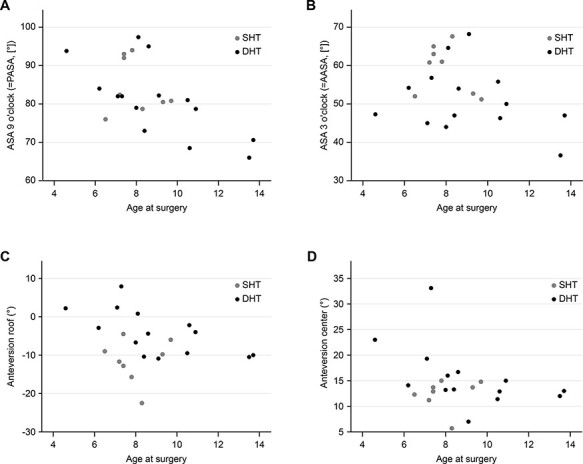
Association of magnetic resonance imaging (MRI)-determined acetabular morphology and patient age at surgery: gray dots represent patients with Spherical Head Types (SHT) (Stulberg (SB) I–II), black dots represent patients with DHT (SB III–V). The horizontal axis depicts the age at surgery in years, and the vertical axis the respective angle in degrees (°). (A) Acetabular Sector Angle (ASA) at 9 o’clock position (posterior ASA (PASA); indicative for coverage of the posterior wall) and (B) ASA at 3 o’clock position (anterior ASA (AASA); indicative for coverage of the anterior wall). (C) Anteversion at the center level and (D) anteversion at the roof level.

There were no trends toward an age-dependent correlation between the age at the time of surgery and the acetabular coverage of the femoral head (Fig. 4).

Furthermore, differentiating between a positive and negative crossover sign showed no significant correlation regarding acetabular version neither just below the roof (−4.7° ± 3.8° versus −7.5° ± 7.4°; *P* = 0.285) nor at the femoral head center (14.5° ± 1.0 versus 14.6° ± 6.2°; *P* = 0.918).

## DISCUSSION

While SIO has the potential to restore the femoral containment and therefore avoid deformity [6, 31–33], several authors have discussed potential overcorrection and acetabular malrotation following this procedure. [34–37]. This was mainly attributed to the displacement of the distal segment of the pelvis in the lateral and anterior directions, which possibly predisposes an acetabular retroversion [38]. Especially in recent years, additional studies have reported on possible acetabular malrotation [38, 39, 40–42]. Their main limitation, however, is the use of plain radiographs [43], which may be influenced by tilt and rotation regarding the presentation of the acetabulum [19]. Simultaneously, we found two studies that used advanced imaging techniques in the assessment of healed LCPD, one by MRI [21] and one by computed tomography (CT) [22]. While a thorough investigation of acetabular version and coverage was performed, these studies did not investigate the sequelae of a specific treatment method but were instead focused on the disease. Therefore, we conducted—to our knowledge—the first study that assessed healed SIO by using MRI.

We found a tendency toward acetabular retroversion both in conventional radiographs and in MRI. In the latter, 15 of 22 acetabula had a version at the femoral head center of less than 15°, which is indicative of a mild retroversion. In their particular subgroup analysis with LCPD patients, Maranho *et al*. [21] found 19 of 21 hips (90%) that had undergone either Salter or triple osteotomy to be retroverted 5 years after study enrollment, while this collective of 21 patients was not further split.

In our study, acetabular version at the femoral head center was not significantly different from the non-operated hips. In contrast, Maranho *et al*. found conservatively treated LCPD patients to have a decreased anteversion when compared with the healthy, contralateral hip [22]. In accordance with Moranho *et al*., however, we found a significantly reduced version just below the acetabular roof [22], who found the difference (7.9°) between LCPD hips and the contralateral hips to be more pronounced at the roof level than at the center level.

In our study, the presence of a crossover sign was not associated with a decrease in MRI-based overall acetabular version. We believe that plain radiographic signs of acetabular retroversion are accentuated due to the effects of SIO at the roof level, while the version at the femoral head center remains uninfluenced.

While several authors found a globally reduced coverage in the FU of LCPD patients [22, 44–46], we found that cases with an osteotomy had an improved anterocranial coverage along with a compromised posterior coverage. Continuous parts of the posterior wall (9, 10 and 11 o’clock positions) were covered significantly less, while most of the anterior wall coverage was similar when compared with the non-operated side.

Noteworthy is the presence of an anterior as well as a posterior impingement sign in 12 and 8 of 22 individuals, respectively, which was significantly more often when compared with the non-operated hips. Although an anterior impingement sign may easily be explained by an increased anterocranial coverage, the posterior impingement sign is not easy to explain. It might also be attributed to sequelae of LCPD on the femoral side, where morphologic changes (i.e. shortened femoral neck and high riding trochanter) can contribute to extra-articular impingement. Regarding a potential correlation between the osteotomy-associated acetabular morphologic changes and patient-related outcomes, it is important to notice that our results show relatively good overall PROM values in the whole cohort. Even the difference in algofunction as well as health-related quality of life between spherical and deformed head types is relatively small. However, as most patients were still very young, deterioration can be expected at least in hips with more severe involvement.

Analyzing age and acetabular remodeling potential may be difficult within our cohort both due to the mean age of 8.5 years at the time of surgery and due to the underlying LCPD. It is known that patients with a late-onset LCPD have poorer outcomes due to the limited femoral head remodeling ability at later ages [1]. Cutoff ages related to possibly poorer outcomes after the age of 4 and 6 years are discussed, depending on the study [41, 47, 48]. In our study, we could not determine an age-related remodeling with regard to acetabular coverage and version (Fig. 4).

Concerning femoral head sphericity, DHT hips were shown to have higher alpha angles and thus a higher degree of femoral head asphericity in comparison to both SHT hips (11, 12, 1 and 2 o’clock) and the non-operated controls (11, 12 and 1 o’clock).

Twenty of 24 hips (83.3%) in our cohort were classified as SB I, II or III hips, implying good to satisfactory radiological results. A multicenter study by Herring *et al*. classified 90% of patients as such [49]. Similarly, 29 of 37 hips (78.4%) in a study by Ishida *et al*. were classified accordingly [10]. Similar results were found in other studies [4, 50]. In a most recent study [13], 26 of 30 hips (86.7%) were SB I, II or III, while 4 were SB IV.

One limitation to our study is the absence of preoperative and direct postoperative MRI, making a continuous FU during adolescence impossible. Therefore, we used the study design by Kobayashi *et al*. [38] comparing the operated hips with the contralateral side. Another limitation is the small sample size. Although the limited number of patients is comparable with similar studies [24, 28, 40, 41], this fact may contribute to the lacking statistical significance of some MRI findings. The majority of patients in our cohort had a concomitant femoral osteotomy, which may affect femoral head containment and potentially the final SB grade. This circumstance may have confounded the results of SIO. Finally, due to costs and time limits, only one MRI Volumetric Interpolated Breath-hold Examination (VIBE) sequence specific for bone morphology was performed, making the assessment of labral and chondral abnormalities not possible. A better determination of the intra-articular status may allow us to predict the future degeneration, since symptomatic patients with sequelae of LCPD may benefit from arthroscopic intervention including chondral or labral repair [51].

## CONCLUSION

In summary, hips treated with SIO were generally not more retroverted at the femoral head center than the non-operated hips while being more retroverted just below the acetabular roof. In addition, there was some remaining dysplasia of the posterior acetabular wall, especially with hips that showed signs of femoral head deformity.

Overall, SIO can lead to satisfying results in patients with LCPD. MRI can help to better understand hip morphologies and the possible causes of emerging hip pain.

## Supplementary Material

hnab063_Supp

## Data Availability

The data underlying this article will be shared on reasonable request to the corresponding author.
