# Supplementary material for: How does former Salter innominate osteotomy in patients with Legg–Calvé–Perthes disease influence acetabular orientation? An MRI-based study
Source: J Hip Preserv Surg. 2021 Aug 21;8(3):240–8. doi: 10.1093/jhps/hnab063 (PMC8994115; doi:10.1093/jhps/hnab063)
Supplement: hnab063_Supp [file hnab063_supp.zip › Suppl_Table_1_JHPS_2021_05_14.docx]

| **Parameter** | **No IVO (n=4)** | **IVO (n=20)** | **P** |
| --- | --- | --- | --- |
| FU_alpha_15 | 31,267 | 48,700 | 0,189 |
| FU_alpha_14 | 46,567 | 67,406 | **0,004** |
| FU_alpha_13 | 65,900 | 65,856 | 0,997 |
| FU_alpha_12 | 55,933 | 63,739 | 0,509 |
| FU_alpha_11 | 61,700 | 50,478 | 0,271 |
| FU_alpha_10 | 50,300 | 40,761 | 0,240 |
| FU_alpha_9 | 33,400 | 32,494 | 0,882 |
| FU_ASA_15 | 47,950 | 55,461 | 0,112 |
| FU_ASA_14 | 69,200 | 92,072 | **0,014** |
| FU_ASA_13 | 111,725 | 118,406 | 0,198 |
| FU_ASA_12 | 111,375 | 119,117 | 0,163 |
| FU_ASA_11 | 103,975 | 110,239 | 0,289 |
| FU_ASA_10 | 94,525 | 94,950 | 0,937 |
| FU_ASA_9 | 81,325 | 82,517 | 0,814 |
| FU_CCA_15 | 28,150 | 31,883 | 0,382 |
| FU_CCA_14 | 60,675 | 62,094 | 0,864 |
| FU_CCA_13 | 81,600 | 76,889 | 0,607 |
| FU_CCA_12 | 78,425 | 65,800 | 0,197 |
| FU_CCA_11 | 64,100 | 68,844 | 0,526 |
| FU_CCA_10 | 59,275 | 55,950 | 0,522 |
| FU_CCA_9 | 45,050 | 48,472 | 0,571 |
| FU_Anteversion_Roof | -2,400 | -7,811 | 0,151 |
| FU_Anteversion_Center | 15,800 | 14,228 | 0,615 |
| FU_LCE | 25,525 | 25,902 | 0,865 |
| FU_AI | 13,700 | 8,747 | 0,232 |
| **Stulberg** |  |  |  |
| *Type I* |  | 6 (25%) |  |
| *Type II* |  | 3 (12,5%) |  |
| *Type III* | 2 (50%) | 11 (45,8%) |  |
| *Type IV* | 1 (25%) | 3 (12,5%) |  |
| *Type V* | 1 (25%) | 1 (4,3%) |  |

**Suppl. Table 1:** Comparison of MRI and xray parameters between group the group that received an intertrochanteric varisation osteotomy (IVO; n=4) and the group that did not. Statistical significance is determined by the Mann-Whitney-U-Test or unpaired t-test, as appropriate. A p-value<.05 indicates statistical significance.
